# The Association Between Fibrosis‐4 Index and All‐Cause and Cardiac Mortality in Patients With Coronary Heart Disease Combined With Diabetes or Prediabetes: Findings From Two Large‐Scale Prospective Cohort Studies

**DOI:** 10.1002/mco2.70786

**Published:** 2026-06-04

**Authors:** Chenxi Song, Zhihao Zheng, Xiaohui Bian, Zheng Qiao, Jiaxi Cheng, Wanqing Sun, Chunyue Wang, Bowen Li, Pengyu Liu, Yuqin He, Rui Fu, Kefei Dou

**Affiliations:** ^1^ Department of Cardiology Fuwai Hospital Chinese Academy of Medical Sciences and Peking Union Medical College Beijing China; ^2^ State Key Laboratory of Cardiovascular Disease National Center for Cardiovascular Diseases Beijing China; ^3^ Emergency Department Inner Mongolia Hospital of Traditional Chinese Medicine Hohhot China; ^4^ Geriatric Department Lanzhou First People's Hospital Lanzhou China; ^5^ Department of Emergency Fuwai Hospital Chinese Academy of Medical Sciences and Peking Union Medical College Beijing China; ^6^ Cardiometabolic Medicine Center Fuwai Hospital Chinese Academy of Medical Sciences and Peking Union Medical College Beijing China

**Keywords:** coronary heart disease, diabetes mellitus, fibrosis‐4 index, mortality, prediabetes, prognosis

## Abstract

Determining the link between fibrosis‐4 index (FIB‐4) score, which serves as a noninvasive metric for identifying liver cirrhosis, and mortality risks from all causes and cardiac causes in individuals with diabetes or prediabetes and confirmed cardiovascular disease remains ambiguous. This study seeks to explore the impact of FIB‐4 on all‐cause and cardiac mortality within this high‐risk clinical group. Two large‐scale patient cohorts were utilized for evaluation: patients from Fuwai Hospital (*N* = 20,133) and the UK Biobank (*N* = 5678). Multivariable Cox regression analysis was employed to estimate hazard ratios (HRs) along with the associated 95% confidence intervals (CIs). In the fully adjusted models, the elevated FIB‐4 category showed a significant association with elevated hazards of all‐cause death (Fuwai Cohort: HR 2.58, 95% CI 1.95–3.42; UKB Cohort: HR 1.26, 95% CI 1.06–1.50) and cardiac mortality (Fuwai Cohort: HR 2.99, 95% CI 1.92–4.66; UKB Cohort: HR 1.41, 95% CI 1.08–1.84). Consistent findings were observed in subgroups based on glucose levels, age, sex, and body mass index. Liver fibrosis, assessed via FIB‐4, showed a significant association with elevated mortality hazards and ought to be incorporated into risk stratification among individuals with coronary heart disease concomitant with either diabetes or prediabetic states.

## Graphical abstract

The current study used two large‐scale cohorts to investigate the association between FIB‐4 index and all‐cause and cardiac‐mortality risk. Elevated FIB‐4, both as categorical and continuous variable, was associated with increased risk of all‐cause and cardiac‐mortality.

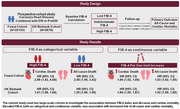

## 1 Introduction

Coronary heart disease (CHD) constitutes a primary driver of global disease burdens, particularly when associated with glycemic abnormalities like diabetes or impaired glucose tolerance. Individuals suffering from CHD alongside diabetes demonstrate a significantly higher likelihood of unfavorable cardiac outcomes and all‐cause mortality, despite receiving ideal pharmacological treatment or invasive procedures [1, 2]. Among those who have already developed atherosclerotic cardiovascular diseases (ASCVD), prediabetes are considered separate risk factors for higher risks of cardiovascular mortality [3]. The ongoing residual risk indicates that there is an urgent necessity to identify new, easily quantifiable prediction indicators so as to achieve more precise risk stratification.

Beyond conventional cardiometabolic indicators, an increasing awareness has emerged regarding the interaction between extra‐cardiac organ impairment and cardiac outcomes. Specifically, the liver—as a pivotal metabolic center—exerts a vital function in systemic inflammation, lipid regulation, and insulin intolerance, each element facilitating atherogenesis plus plaque vulnerability [4]. Metabolic dysfunction‐driven steatotic liver disorder (MASLD) represents a unique risk element that substantially raises the probability of major adverse cardiovascular events and leads to elevated hazards of cardiac‐associated death [5, 6]. This liver–heart axis emphasizes the possible value of hepatic fibrosis evaluation in cardiovascular risk estimation.

Despite liver biopsy serving as the histological benchmark for fibrosis staging, this invasive technique involves significant expenses and is unfeasible for extensive screening or routine follow‐up within cardiovascular subjects [7]. Therefore, noninvasive serum‐focused metrics have been established and confirmed. Specifically, the fibrosis‐4 index (FIB‐4) score, derived from patient age alongside levels of aspartate aminotransferase (AST), alanine aminotransferase (ALT), and platelet enumeration, has become a basic, affordable, and broadly available instrument. FIB‐4 scores demonstrate powerful diagnostic accuracy for detecting severe fibrosis throughout various groups, including people suffering from diabetes and MASLD [8, 9]. Recognizing its medical importance, existing recommendations from the American Diabetes Association (ADA) suggest utilizing FIB‐4 to identify advanced hepatic fibrosis among patients with confirmed ASCVD who also had diabetes or prediabetes [10].

While it is widely acknowledged that the FIB‐4 index functions as a robust indicator for liver‐associated clinical outcomes, its specific prognostic significance regarding hard outcomes—namely, all‐cause and cardiac mortality, in high‐risk cardiometabolic groups remains poorly established. Recent studies have found that a high FIB‐4 score is associated with an increased risk of cardiac events across diverse cohorts and individuals with nonalcoholic fatty liver disease (NAFLD) [11, 12]. Nevertheless, substantial long‐term investigations focusing explicitly on subjects with confirmed CHD and dysglycemia—an entity characterized by intense metabolic and cardiovascular vulnerability—are currently scarce.

Thereafter, the main purpose of this study is to reveal the relationship between baseline FIB‐4 values and risks for all‐cause and cardiac mortality among a high‐risk population with CHD who were simultaneously diagnosed with diabetes or prediabetes at admission. Based on the two large‐scale prospective cohort studies of the Fuwai Hospital in China and the UK Biobank, we explored whether FIB‐4 could add more prognostic value in addition to traditional risk factors, to support its inclusion in potential risk assessment for future cardiac metabolic management

## 2 Results

### 2.1 Baseline Characteristics

**FIGURE 1:**
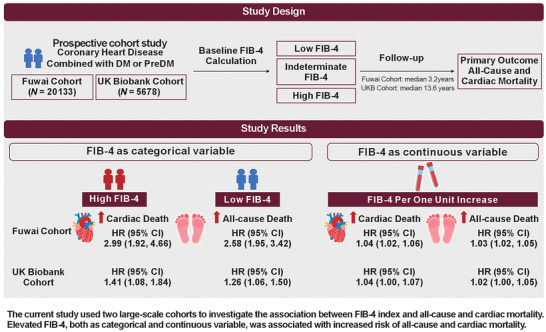
The current study used two large‐scale cohorts to investigate the association between FIB‐4 index and all‐cause and cardiac mortality. Elevated FIB‐4, both as categorical and continuous variable, was associated with increased risk of all‐cause and cardiac mortality. CI, confidence interval; FIB‐4, fibrosis‐4 index; HR, hazard ratio.

**FIGURE 2:**
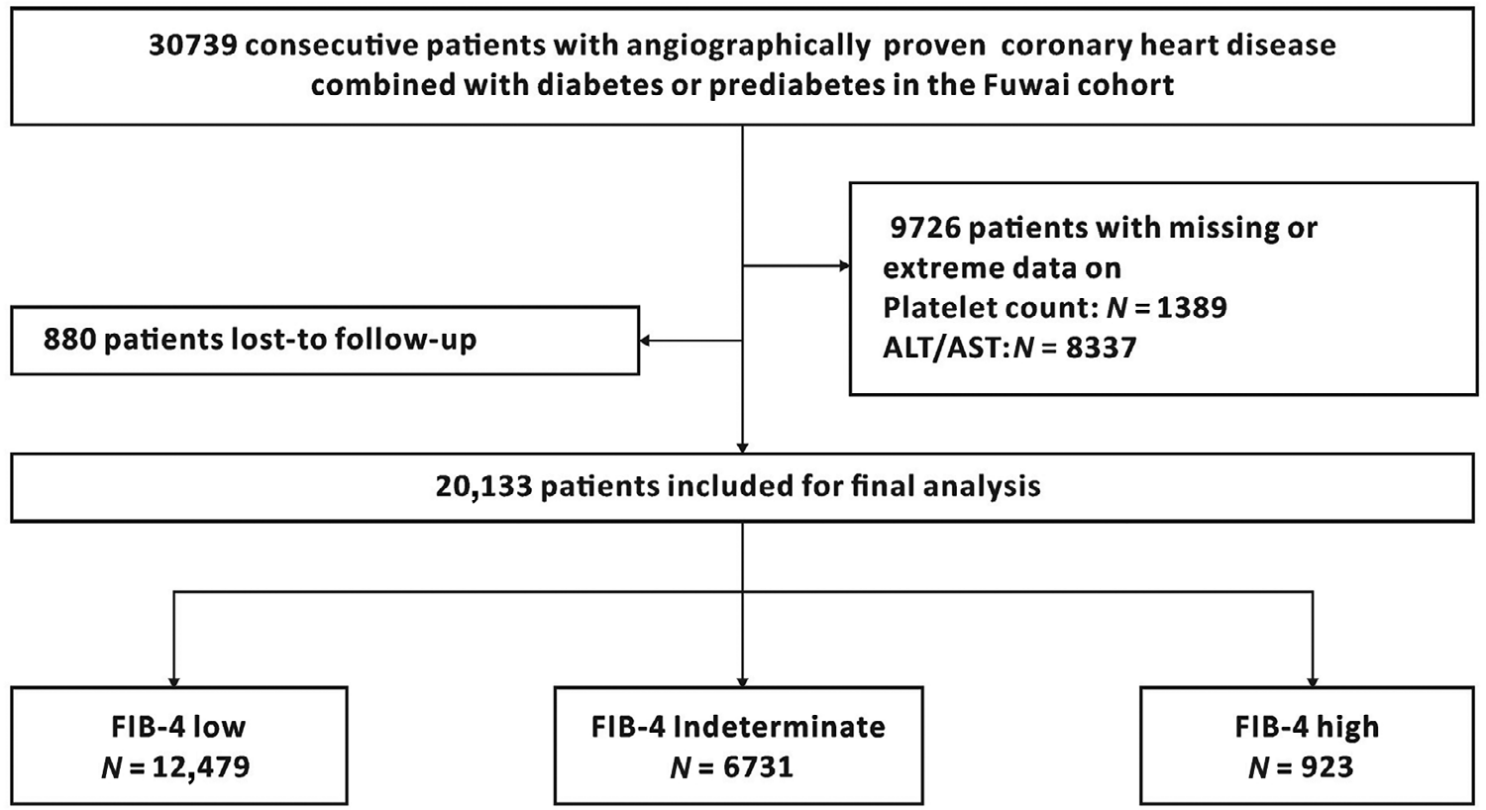
Study flow chart in the Fuwai Cohort. A total of 30,739 patients who underwent angiography from January 2017 to December 2018, and diagnosed coronary heart disease combined with diabetes or prediabetes were screened. After the exclusion of 9726 patients with missing data on platelet count, ALT or AST, and 880 patients with lost‐to follow‐up, a total of 20,133 patients were included for final analysis. ALT, alanine aminotransferase; AST, aspartate aminotransferase; FIB‐4, fibrosis‐4 index.

The general research framework and primary findings are depicted in Figure 1. To sum up, based on information from two extensive, prospective, observational cohort programs: The Fuwai Cohort and the UK Biobank Cohort, we investigate how the baseline FIB‐4 score relates to long‐term survival in patients with CHD coexisting with either diabetes or prediabetes. Regarding the Fuwai Cohort, a total of 20,133 individuals were incorporated into the ultimate evaluation. The median age of the study cohort was 61 years, and 26.3% represented females. The participant recruitment flow chart for the Fuwai Cohort is illustrated in Figure 2. Baseline characteristics are detailed in Table 1. The frequency distribution of FIB‐4 scores is presented in Figure S1. The median FIB‐4 was 1.27 (interquartile range [IQR] 0.92–1.72). Compared with the low FIB‐4 group, patients in the high FIB‐4 group exhibited significantly elevated age, glycated hemoglobin (HbA1c), and high‐sensitivity C‐reactive protein (hsCRP) levels. The frequency of previous myocardial infarction (MI) was likewise higher in the high FIB‐4 group. The levels of body mass index (BMI) and serum triglycerides (TG) were lower in the high FIB‐4 group.

**TABLE 1:** Baseline characteristics according to baseline FIB‐4 category in the Fuwai Cohort.

Characteristics	FIB‐4 low *N* = 12,479	FIB‐4 indeterminate *N* = 6731	FIB‐4 high *N* = 923	*p* value
Age, years	57.27 ± 9.05	65.99 ± 8.34	66.07 ± 11.29	<0.0001
Male, *n* (%)	9501/12,479 (76.14)	4670/6731 (69.38)	665/923 (72.05)	<0.0001
BMI, kg/m^2^	26.28 ± 3.25	25.54 ± 3.16	25.37 ± 3.69	<0.0001
Current smokers, *n* (%)	4519/12,479(36.21)	1777/6731(26.40)	276/923(29.90)	<0.0001
Hypertension, *n* (%)	8089/12,479(64.82)	4450/6731(66.11)	546/923(59.15)	0.0001
SBP, mmHg	130.29 ± 17.22	132.23 ± 18.40	124.49 ± 19.70	<0.0001
DBP, mmHg	78.30 ± 10.94	76.14 ± 10.88	74.84 ± 12.26	<0.0001
Prior MI, *n* (%)	2696/12,479(21.60)	1523/6731(22.63)	396/923(42.90)	<0.0001
Prior stroke, *n* (%)	1482/12,479(11.88)	1041/6731(15.47)	102/923(11.05)	<0.0001
**Biochemical parameters**				
ALT(U/L)	25.00(18.00, 37.00)	20.00(14.00, 30.00)	31.00(20.00, 53.00)	<0.0001
AST(U/L)	21.00(17.00, 26.00)	24.00(20.00, 30.00)	86.00(40.00, 166.00)	<0.0001
PLT(10^9^/L)	244.00(212.00, 284.00)	191.00(164.00, 219.00)	175.00(140.00, 220.00)	<0.0001
TC, mmol/L	4.11 ± 1.08	4.06 ± 1.05	4.03 ± 1.03	0.0013
HDL‐C, mmol/L	1.08 ± 0.28	1.17 ± 0.32	1.13 ± 0.34	<0.0001
LDL‐C, mmol/L	2.49 ± 0.91	2.40 ± 0.89	2.44 ± 0.89	<0.0001
TG, mmol/L	1.53(1.15, 2.13)	1.39(1.04, 1.92)	1.27(0.93,1.78)	<0.0001
Lp(a), mg/L	166.59 (70.00, 410.00)	163.17 (68.00, 398.69)	174.39 (75.88, 378.00)	0.3180
ApoA1, g/L	1.36 ± 0.27	1.42 ± 0.29	1.30 ± 0.30	<0.0001
ApoB, g/L	0.78 ± 0.23	0.74 ± 0.22	0.76 ± 0.24	<0.0001
FPG, mmol/L	6.65 ± 1.22	6.52 ± 1.13	6.55 ± 1.44	0.0234
HbA1c, %	6.87 ± 2.54	6.77 ± 2.48	8.54 ± 3.93	<0.0001
hsCRP, mg/L	1.53(0.75, 3.24)	1.40(0.67,3.02)	3.38(1.22,11.07)	<0.0001
Creatinine, µmol/L	79.59(70.13, 90.12)	80.80(70.31, 92.63)	79.22(67.69, 93.30)	0.4480
**Medication at discharge**				
Antidiabetic drugs (%)	5033/12,479(40.33)	2495/6731(37.07)	333/923(36.08)	<0.0001
Statins, *n* (%)	12,141/12,479(97.29)	6510/6731(96.72)	903/923(97.83)	0.0315

FIB‐4 index was categorized as low (<1.45), indeterminate (1.45–3.25), and high (>3.25).

Abbreviations: ALT, alanine aminotransferase; ApoA1, apolipoprotein A1; ApoB, apolipoprotein B; AST, aspartate aminotransferase; BMI, body mass index; DBP, diastolic blood pressure; FIB‐4, fibrosis‐4 index; FPG, fasting plasma glucose; HbA1c, glycated hemoglobin; HDL‐C, high‐density lipoprotein cholesterol; hsCRP, high‐sensitivity C‐reactive protein; LDL‐C, low‐density lipoprotein cholesterol; Lp(a), lipoprotein (a); MI, myocardial infarction; PLT, platelet; SBP, systolic blood pressure; TC, total cholesterol; TG, triglycerides.

Regarding the UK Biobank Cohort, 5678 individuals were ultimately incorporated into the final evaluation. The median age of the study cohort was 63 years, while 30.35% represented females. Baseline characteristics are summarized in Table S1. The distribution frequency of FIB‐4 scores is illustrated in Figure S2. The median FIB‐4 figure stood at 1.44 (IQR 1.14–1.89). Compared with the low FIB‐4 group, levels of age and the concentration of C‐reactive protein (CRP) were significantly higher in the high FIB‐4 cohort, and there existed a tendency toward a greater proportion of previous MI within the high FIB‐4 category.

### 2.2 Clinical Outcomes by Baseline FIB‐4 Index

Throughout a median follow‐up period of 3.2 years (IQR 3.0–3.5 years), a cumulative total of 542 all‐cause deaths and 210 cardiovascular deaths were recorded within the Fuwai Cohort. The proportion of all‐cause mortality observed among patients stratified into low‐, indeterminate‐, and high‐risk FIB‐4 categories were 1.67, 3.64, and 9.64%, respectively. Furthermore, the proportion of cardiac mortality for these same risk categories were 0.63, 1.41, and 3.90%, respectively.

Regarding the UK Biobank Cohort, throughout a median follow‐up of 13.6 years (IQR 10.3–13.5 years), a cumulative total of 1933 all‐cause deaths and 782 cardiac deaths took place. Corresponding with findings from the Fuwai Cohort, the incidence of all‐cause and cardiac death rose progressively throughout the low‐, indeterminate‐, and high‐risk FIB‐4 groups.

**FIGURE 3:**
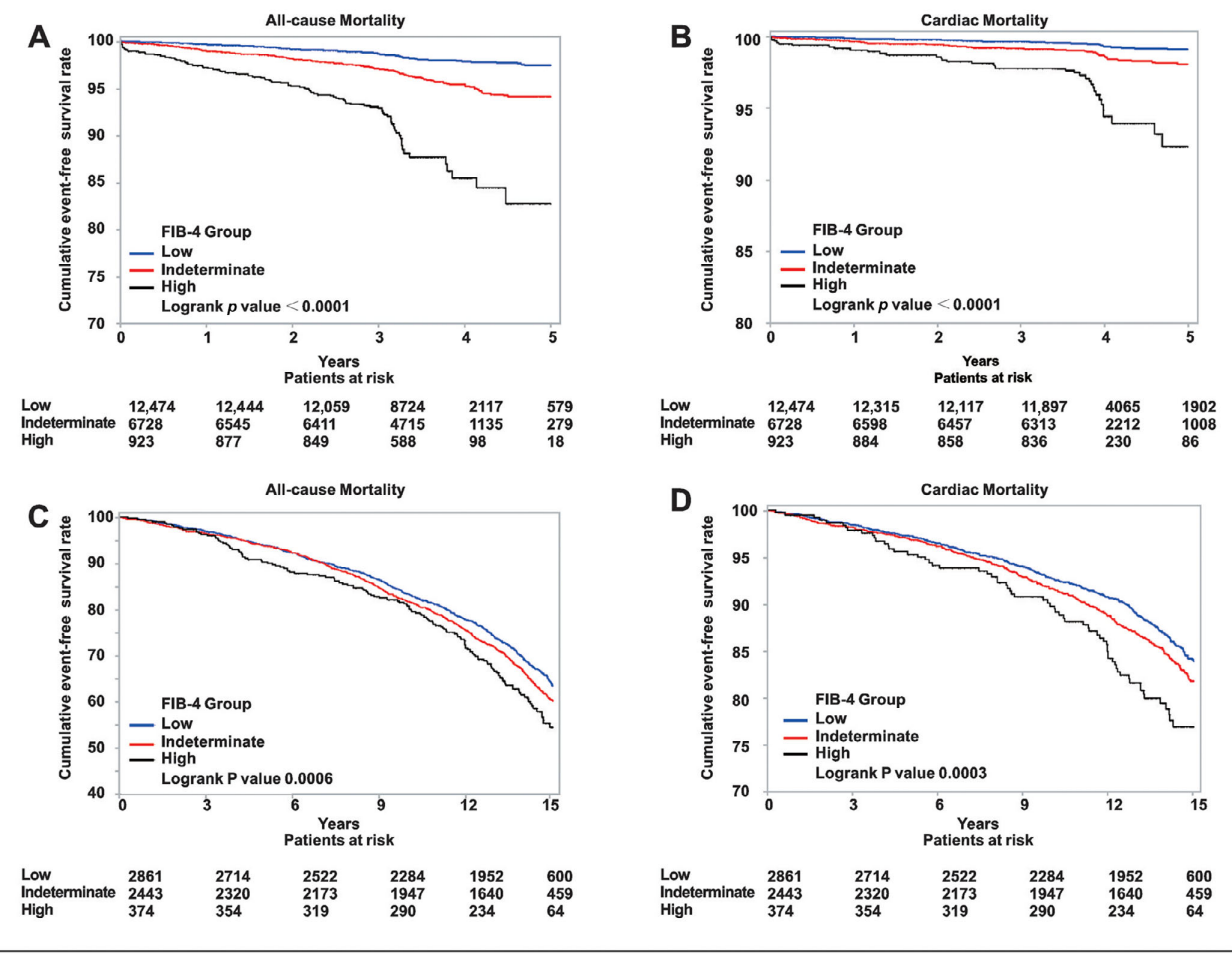
The cumulative event‐free survival analysis according to the FIB‐4 index group. Kaplan–Meier curves showing the all‐cause mortality‐free survival rate and cardiac death‐free survival rate in the Fuwai Cohort (A and B) and the UK Biobank Cohort (C and D). FIB‐4, fibrosis‐4 index.

The Kaplan–Meier curves regarding FIB‐4 stratification are displayed in Figure 3 for all‐cause and cardiac mortality. Within both the Fuwai (Figure 3A,B) and UK Biobank Cohort (Figure 3C,D), the cumulative incidence for both adverse events were highest in the high FIB‐4 group and lowest in the low FIB‐4 group. Regarding the Fuwai Cohort, individuals within the high FIB‐4 category exhibited a greater risk of all‐cause mortality (adjusted hazard ratio [HR]: 2.584, 95% confidence interval [CI]: 1.954–3.417, *p* < 0.0001) and cardiac mortality (adjusted HR: 2.989, 95% CI: 1.919–4.656, *p* < 0.0001) relative to the low FIB‐4 group, following correction for possible confounders (Table 2). Every one‐unit increment in the FIB‐4 score corresponded to a 3.3 and 4.1% increased likelihood of death from any cause as well as from cardiac causes, respectively. Further adjustments for statins, previous MI, left ventricular ejection fraction (LVEF), and triple‐vessel disease during angiography produced consistent findings (Table S2). For the UK Biobank Cohort, subjects in the high FIB‐4 category showed an elevated likelihood of death from any cause (adjusted HR: 1.261, 95% CI: 1.058–1.504, *p* = 0.0098) and cardiac mortality (adjusted HR: 1.407, 95% CI: 1.079–1.836, *p* = 0.0118) compared with the low FIB‐4 group, following adjustment for potential confounders (Table S3). Further adjustments for statins, previous MI, alcohol consumption, and hepatotoxic medications yielded similar results (Table S4).

**TABLE 2:** The association between FIB‐4 with all‐cause and cardiac mortality in the Fuwai Cohort.

	Event/total (%)	Crude HR	*p* value	Adjusted HR	*p* value
**All‐cause mortality**					
FIB‐4 low	208/12,479 (1.67)	1 (ref)	1 (ref)	1 (ref)	1 (ref)
FIB‐4 indeterminate	245/6731 (3.64)	2.212 (1.838, 2.661)	<0.0001	1.201 (0.981, 1.471)	0.0762
FIB‐4 high	89/923 (9.64)	6.408 (4.997, 8.218)	<0.0001	2.584 (1.954, 3.417)	<0.0001
FIB‐4 per one unit increase	542/20,133 (2.69)	1.082 (1.072, 1.093)	<0.0001	1.033 (1.020, 1.047)	<0.0001
**Cardiac mortality**					
FIB‐4 low	79/12,479 (0.63)	1 (ref)	1 (ref)	1 (ref)	1 (ref)
FIB‐4 indeterminate	95/6731 (1.41)	2.234 (1.658, 3.011)	<0.0001	1.248 (0.901, 1.729)	0.1821
FIB‐4 high	36/923 (3.90)	6.888 (4.642, 10.22)	<0.0001	2.989 (1.919, 4.656)	<0.0001
FIB‐4 per one unit increase	210/20,133 (1.04)	1.086 (1.071, 1.101)	<0.0001	1.041 (1.022, 1.061)	<0.0001

Adjusted for age, sex, current smoking status, prior hypertension, body mass index, low‐density lipoprotein, cholesterol, estimated glomerular filtration rate, and HbA1c.

FIB‐4 index was categorized as low (<1.45), indeterminate (1.45–3.25), and high (>3.25).

Abbreviations: CI, confidence interval; HR, hazard ratio; FIB‐4, fibrosis‐4 index.

**FIGURE 4:**
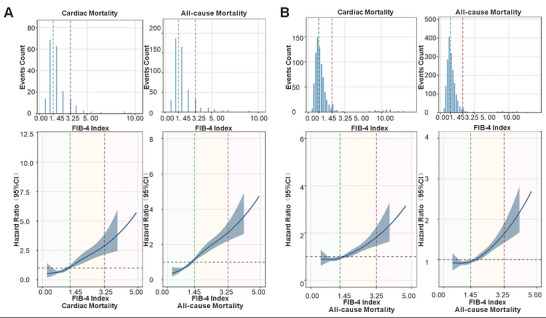
Restricted cubic spline of all‐cause and cardiac mortality and FIB‐4 index. (A) Fuwai Cohort; (B) UK Biobank Cohort. FIB‐4, fibrosis‐4 index.

Cubic spline functions exhibited a positive linear correlation between FIB‐4 indices and the hazard of all‐cause and cardiac mortality within the Fuwai Cohort (Figure 4A) alongside the UK Biobank Cohort (Figure 4B).

### 2.3 Sensitivity and Subgroup Analysis

To further evaluate these correlations, sensitivity analyses were performed by applying a different threshold for FIB‐4. These findings demonstrated that the multivariate‐adjusted HRs for the high FIB‐4 category relative to the low category was 1.854 (95% CI: 1.414–2.430) for all‐cause mortality and 1.988 (95% CI: 1.292–3.060) regarding cardiac mortality within the Fuwai Cohort (Table S5). Comparable results were identified in the UK Biobank Cohort, as individuals in the high FIB‐4 category displayed a greater risk of all‐cause and cardiac mortality than those in the low FIB‐4 category (Table S6).

Sensitivity analyses employing the prediabetes definitions based on the World Health Organization (WHO) standards or omitting individuals experiencing outcomes during the initial 6 months produced no substantially divergent outcomes from the main findings across these two cohorts (Tables S7–S10).

Subgroup analyses investigating the links between FIB‐4 and cardiac mortality within both cohorts are detailed in Tables S11 and S12. Broadly, the HR values for cardiac mortality were elevated in the high FIB‐4 category relative to the low FIB‐4 group throughout every subgroup. These correlations stayed stable in subgroups by glycemic conditions (diabetes or prediabetes), age (age <65 and ≥65 years), gender, and BMI (BMI < 25 kg/m^2^ or ≥25 kg/m^2^), with interaction *p* values exceeding 0.05.

## 3 Discussion

This present research utilized datasets from two extensive cohorts, the Fuwai Cohort and the UK Biobank Cohort, to investigate the impact of the FIB‐4 score on the risks of all‐cause and cardiac mortality. Results from both cohorts indicated that elevated FIB‐4, evaluated as a categorical or continuous variable, was linked to a heightened risk of all‐cause and cardiac mortality following the adjustment for conventional cardiovascular risk factors and HbA1c. Such connections persistent across subgroups categorized by glucose levels, age, gender, and BMI. Our results imply that hepatic fibrosis, as measured by the FIB‐4 index, should be considered in individuals suffering from CHD concomitant with diabetes or prediabetes.

### 3.1 Different Results Between the Fuwai and UKB Cohort

The median length of follow‐up among patients from the Fuwai Cohort (3.2 years) differs significantly from that of the UK Biobank Cohort (13.6 years). The extension of the follow‐up in the UK Biobank can help identify subsequent events to improve analysis of long‐term effects. However, the shorter length of follow‐up in the Fuwai Cohort reflects a more acute clinical setting following coronary angiography. Importantly, the good correspondence of the association between elevated FIB‐4 and mortality in both short‐ and long‐term follow‐up periods verifies that the FIB‐4 index can be used as an initial and persistent risk indicator. The difference in observation time may be one reason for the lower absolute incidence rates, yet this has no impact on whether there exists an independent association.

Numerically, the mortality hazard ratio (HR) for elevated FIB‐4 was higher in the Fuwai Cohort than that of the UK Biobank Cohort at a specific level. Multiple elements likely account for this discrepancy. The Fuwai Cohort was composed of patients who underwent coronary angiography or percutaneous coronary intervention. Thus, it included a greater proportion of highly selected and actively treated patients with cardiovascular diseases than the UK Biobank. Additionally, variations across ethnicity, habits, and healthcare systems affect the perceived effect magnitude differently. There are also differences in the laboratory test methods used to evaluate the FIB‐4 index and differences in sampling time (before angiography versus initial enrollment). However, the statistically significant correlation we found apply to both cohorts, supporting the broad applicability of our findings.

### 3.2 Comparison With Previous Studies

The FIB‐4 score was initially introduced to evaluate hepatic fibrosis in patients concurrently infected with human immunodeficiency virus and hepatitis C virus [8]. Subsequent researches have confirmed the diagnostic value of the FIB‐4 metric in patients with the diagnosis of MASLD, and its prognostic capacity to identify individuals at risk for subsequent cirrhosis or liver‐related events among the general population, patients with NAFLD, or people with obesity or diabetes [13, 14, 15, 16, 17, 18].

Beyond hepatic outcomes, a rising awareness exists regarding the impact of FIB‐4 index on cardiovascular endpoints. This particular index has been connected to unfavorable cardiac outcomes within the general population, as well as unfavorable cardiac outcomes in subjects with obesity, diabetes, or NAFLD [14, 18, 19, 20]. A recent systematic review indicated that heightened FIB‐4 values correlated with a higher probability of cardiac mortality and poor outcomes in individuals suffering from cardiovascular diseases, such as CHD and congestive heart failure [21]. Nevertheless, the impact of FIB‐4 measurement on the likelihood of deaths of all‐cause and cardiac causes in patients exhibiting CHD alongside diabetes mellitus or prediabetic conditions remains undefined.

### 3.3 Our Findings and Possible Mechanisms

This research employed two extensive cohorts to explore the correlation between the FIB‐4 score and the hazard of all‐cause and cardiac mortality. Following correction for HbA1c and various conventional cardiovascular risk elements, the high FIB‐4 group exhibited a heightened probability of all‐cause and cardiac mortality relative to the low FIB‐4 group across both cohorts. When the FIB‐4 metric was regarded as a continuous variable, every one‐point rise in the FIB‐4 value remained substantially linked with a heightened risk of all‐cause and cardiac mortality. Subgroup analyses indicated that glycemic status (diabetes or prediabetes) did not modify these correlations, and the correlations stayed stable within both acute coronary syndrome and chronic coronary syndrome categories, which matches prior study findings [22, 23]. These results indicate that the elevated mortality risk resulting from advanced hepatic fibrosis likely involves mechanisms distinct from glucose regulation and conventional risk factors.

While specific mechanisms behind these correlations remain unclear, possible pathways involve heightened systemic inflammation plus cardiac impairment [24, 25]. Hepatic cirrhosis is intimately linked with intensified systemic inflammation, which serves as a primary catalyst for atherosclerotic advancement and vulnerable plaque disruption, leading to increased cardiovascular mortality [26, 27]. Our initial findings also indicate that the high FIB‐4 group exhibited increased levels of inflammatory indicators like hsCRP, relative to the low FIB‐4 group. Furthermore, liver cirrhosis can negatively influence both cardiac diastolic and systolic function, a condition known as cirrhotic cardiomyopathy, which significantly impacts patients’ general cardiovascular wellness and clinical outcomes [28]. Liver fibrosis might aggravate myocardial structural remodeling and malfunction via systemic inflammation, oxidative stress, metabolic disturbances, and coronary microvascular impairment [29, 30].

### 3.4 Clinical Significance

Existing recommendations by the ADA suggest employing the FIB‐4 score for screening diabetic or prediabetic individuals with confirmed cardiovascular diseases to assess their likelihood of progressing to cirrhosis linked with metabolic dysfunction‐associated steatohepatitis [31]. Our results reinforce this suggestion from an alternative viewpoint, since a raised FIB‐4 value correlated with a higher hazard of all‐cause and cardiac mortality. Such correlations stayed significant following the correction for conventional cardiovascular risk variables and glucose metrics, underscoring the predictive utility of the FIB‐4 measure. The capacity to forecast mortality risk via the FIB‐4 tool facilitates earlier risk categorization and possibly more intensive therapeutic approaches.

Regarding health economics, FIB‐4 only requires basic blood tests including age, serum AST/ALT levels, and platelets and thus serves as an economical instrument. Incorporating this metric into current clinical routines requires negligible additional costs and might promote early risk stratification and intensified treatment of patients with elevated risks.

### 3.5 Strengths and Limitations

A primary strength of this study involves using two large‐scale clinical cohort data, thus verifying the robustness of the conclusions of the association between FIB‐4 index and mortality risk. However, some limitations arise from the current study. First, while accounting for an extensive array of clinically significant covariates, which included age, sex, BMI, current smoking status, hypertension, lipid levels, renal status, and glycemic management, the likelihood of residual confounding remains unconfirmed. Unmeasured or imprecisely quantified variables like physical exercise, dietary patterns, medication adherence, genetic predisposition, or environmental exposures might affect liver fibrosis as well as survival outcomes. Even though several sensitivity and subgroup analyses demonstrated stable correlations, definitive causal conclusions must be drawn carefully. Subsequent prospective research incorporating thorough phenotyping and potentially mendelian randomization techniques would facilitate the clarification of these relationships. Additionally, only baseline FIB‐4 values were accessible, meaning the connection between variations in this index and mortality hazards was unexplored. Furthermore, details concerning the administration of hepatotoxic medications or alcohol intake were lacking in the Fuwai Cohort. Consequently, we were unable to identify individuals with chronic liver conditions caused by pharmacological or alcoholic use. Ultimately, racial discrepancies in the prevalence, clinical presentation, and histopathological characteristics of hepatic fibrosis persist. Hence, the generalizability of our conclusions to diverse study groups necessitates subsequent confirmation.

## 4 Conclusions

Severe hepatic fibrosis, categorized through an elevated FIB‐4 score, linked to an increased likelihood of all‐cause and cardiac mortality among individuals suffering from CHD alongside diabetes or prediabetes. These results endorse the current recommendation to utilize the FIB‐4 metric as the first‐line index for risk stratification in this vulnerable cohort, by illustrating the prognostic utility of the FIB‐4 tool. Since such correlations remained unaffected after the adjustment of conventional cardiac risk factors, subsequent investigations into the fundamental pathophysiology are essential.

## 5 Materials and Methods

Based on two extensive, long‐term observational cohorts—namely, the Fuwai Cohort and the UK Biobank Cohort, this research aimed to investigate how baseline FIB‐4 value affects patients’ likelihood of all‐cause and cardiac mortality over time. Ethical approval for the Fuwai Cohort was granted by the Ethics Board of Fuwai Hospital (2016‐847), and the project followed the guidelines of the Declaration of Helsinki. Prior to enrollment, every participant provided written informed consent under protocol version AS2016‐1.1. Ethical approval for the UK Biobank investigation was approved by the North West Multicenter Research Ethics Committee (REC number: 11/NW/0382), and formal written consent was provided by every participant. This examination utilized UK Biobank information through Application 97155.

### 5.1 Study Population

This research utilized information from two extensive patient cohorts with CHD, specifically the Fuwai Cohort and the UK Biobank Cohort.

*Fuwai Cohort*: The schematic diagram for participant recruitment is shown in Figure 2. In brief, we consecutively recruited individuals older than 18 years who received angiography and received a diagnosis of CHD, while also fulfilling prerequisites for diabetes or prediabetes. CHD was defined as clinical manifestations of angina or dyspnea, combined with one or more stenoses exceeding 50% in major coronary arteries as identified via coronary angiography. Diabetes was identified according to documented medical history, the administration of hypoglycemic drugs, fasting plasma glucose (FPG) reach or exceed 7.0 mmol/L, or glycated hemoglobin (HbA1c) value ≥6.5%. Prediabetes was defined using the ADA standards: HbA1c value between 5.7 and 6.4%, or a FPG levels ranging from 5.6 to 6.9 mmol/L, without any diabetes medical history or the antidiabetic treatment. Subjects with a diagnosis of viral hepatitis were excluded from the study. Altogether, 30,739 consecutive subjects with angiographically verified CHD alongside with diabetes or prediabetes were evaluated. Among these, 9726 patients were excluded owing to insufficient information regarding AST, ALT, or platelet enumeration, and 880 patients were removed because of loss‐to follow‐up. The ultimate cohort comprised 20,133 subjects.

*UK Biobank Population*: The schematic diagram illustrating participant recruitment is presented in Figure S3. This study employs data from the UK Biobank, acknowledged as the most extensive publicly available dataset for medical investigation. This large‐scale prospective cohort includes more than 500,000 individuals ranging from 40 to 69 years during initial enrollment throughout the United Kingdom, and the gathered information includes questionnaires, physical measurements, biochemical specimens, and imaging scans [32, 33]. Subjects' records were connected to National Health Service records, such as hospital admissions, death registry data, and cancer registry data, enabling the detection of prior‐enrollment CHD medical history and tracking of subsequent‐follow‐up adverse outcomes.

Eligibility criteria required the existence of CHD alongside diabetes or prediabetes before participation. CHD was identified via self‐reported medical‐confirmed angina or heart attack or hospital documentation based on ICD‐10 marked CHD diagnoses. Diabetes was defined by a clinician during the touchscreen survey, insulin and antihyperglycemic drug consumption, FPG values ≥ 7.0 mmol/L, or HbA1c levels ≥ 6.5%. Prediabetes was characterized by ADA guidelines: HbA1c values from 5.7 to 6.4%, or FPG values from 5.6 to 6.9 mmol/L, in the absence of diabetes medical history or diabetic medication use. We identified a total of 8870 participants with CHD plus diabetes or prediabetes, excluding 723 individuals with chronic liver disease or alcohol‐related conditions, 770 subjects with cancer, and 1699 cases with insufficient data for ALT, AST, or platelet count (PLT), plus PLT ≤ 30 × 10^9^/L, ultimately enrolling 5678 subjects for evaluation.

The specific identifiers for primary variables are listed in Table S13, and each diagnostic date was confirmed to precede the actual enrollment date.

### 5.2 Fibrosis Score Measurement

Regarding the Fuwai Cohort, venous blood was sampled before undergoing angiography. A full blood cell count and standard biochemical assessments were executed systematically to evaluate the concentrations of AST, ALT, and PLT. Each laboratory evaluation was carried out at the biochemical laboratory of Fuwai Hospital following a standardized procedure. Concerning the UK Biobank Cohort, plasma was obtained during the initial enrollment stage. The level of AST and ALT were analyzed utilizing a Beckman Coulter AU5800, while the platelet quantity was assessed through a hematology testing method.

FIB‐4 was computed as: age (years) × (AST [U/L])/(platelets [10^9^/L] × [ALT]^1/2^). Patients were categorized into three distinct groups based on the baseline FIB‐4 cutoff values proposed by Sterling et al. [8], specifically <1.45, 1.45–3.25, and >3.25 for those belonging to the low‐, intermediate‐, and high‐risk FIB‐4 group.

$$\mathrm{FIB}-4=\frac{\mathrm{age}\;\left(\mathrm{years}\right)\times \mathrm{AST}\left(U/L\right)}{\sqrt{\mathrm{ALT}\;\left(U/L\right)}\times \mathrm{platelet}\;\mathrm{count}\;\left(10^{9}/L\right)}$$

### 5.3 Follow‐up and Outcome

The follow‐up of the Fuwai Cohort was conducted by professional cardiologists at 3 years postdischarge via telephone contact or on‐site visits. The primary outcome were all‐cause mortality and cardiac mortality. Cardiac mortality refers to death due to cardiac causes, including sudden cardiac arrest, acute MI, heart failure, strokes, and related cardiovascular diseases. Undetermined deaths were classified as cardiac mortality during outcome determination. Every outcome event was independently confirmed by at least two different cardiologists.

Regarding the UK Biobank Cohort, the outcome data were retrieved via integration with death registries. All‐cause mortality was verified whenever the specific date of death was documented. Cardiac mortality was defined as death linked to the circulatory system based on ICD‐10 codes (I00–I99). At the time of analysis, clinical status data remained accessible until December 30, 2023. During data analysis, health outcome records were accessible until December 30, 2023. This date served as the censoring point for follow‐up in our study, except in cases where the primary endpoint occurred prior to this date.

### 5.4 Statistical Analysis

Baseline continuous variables were reported as means ± standard deviations or medians with IQR, whereas categorical variables were reported as counts (proportions). Continuous variables were compared using analysis of variance or the Mann–Whitney *U* test, and categorical data were compared using the chi‐square test. The Bonferroni adjustment technique was employed to modify the *p* values by partitioning the desired alpha threshold (*p* = 0.05) by the total number of examinations performed (*n* = 3).

Kaplan–Meier methods were utilized to calculate the total event‐free survival probabilities for various FIB‐4 groups, which were subsequently contrasted via log‐rank tests. We employed Cox proportional hazards regression models to estimate the HRs along with their corresponding 95% CIs, taking the low FIB‐4 group (<1.45) as the reference group. Both unadjusted and adjusted HR values were calculated. The proportional hazards assumption was verified through the application of Martingale‐based residual assessments (Figures S4 and S5). A set of clinical variables were incorporated into the Cox proportional hazards model, which included age, sex, BMI, current smoking status, hypertension, low‐density lipoprotein cholesterol, and estimated glomerular filtration rate. Supplementary confounders considered for adjustment comprised statin therapy, previous MI, LVEF, triple‐vessel coronary artery disease, alcohol consumption, and hepatotoxic pharmacological agents. Hepatotoxic drugs were identified as methotrexate, amiodarone, tamoxifen, fluorouracil, irinotecan, carboplatin, cisplatin, and oxaliplatin, based on prior research [18]. The relationship between the FIB‐4 metric as a continuous parameter and all‐cause mortality or cardiac mortality was investigated utilizing restricted cubic spline modeling.

Multiple sensitivity evaluations were conducted: First, the link between mortality and FIB‐4 was examined utilizing distinct FIB‐4 cutoffs stratifying patients into low (<1.30), intermediate (1.30–2.67), and high (>2.67) risk categories. Subsequently, prediabetes was categorized based on WHO/International Expert Committee standards: HbA1c levels between 6.0 and 6.4%, or FPG levels between 6.1 and 6.9 mmol/L, while excluding individuals with diabetes history or current diabetic therapy. Ultimately, to assess potential reverse causality, separate analyses omitting subjects who died within the initial 6‐month period were performed.

The proportion of missing data was minimal for each covariate within both cohorts, and missing data were imputed using the average or median of the recorded figures (Table S14). All statistical analyses was conducted utilizing SAS software (SAS Institute, USA).

## Author Contributions

**Chenxi Song**: data curation, formal analysis, methodology, and writing – original draft. Zhihao Zheng: data curation, software, and writing – review and editing. **Xiaohui Bian**: data curation and formal analysis. **Zheng Qiao**: formal analysis and writing – original draft. **Jiaxi Cheng**: visualization, validation, methodology, and investigation. **Wanqing Sun**: data curation, formal analysis, methodology, and writing – original draft. **Chunyue Wang**: data curation, formal analysis, methodology, and writing – review and editing. **Bowen Li**: data curation, software, and writing – review and editing. **Pengyu Liu**: validation and writing – review and editing. **Yuqin He**: writing – review and editing. **Rui Fu**: conceptualization, funding acquisition, supervision, and writing – review and editing. **Kefei Dou**: conceptualization, funding acquisition, supervision, and writing – review and editing. The final version of the manuscript has been reviewed and endorsed by all contributing authors.

## Funding

The research was sponsored by the Noncommunicable Chronic Diseases‐National Science and Technology Major Project (2025ZD0548200), Noncommunicable Chronic Diseases‐National Science and Technology Major Project (2024ZD0539300), and the National Natural Science Foundation of China (No. 3230050498).

## Ethics Statement

Permission to conduct the Fuwai Cohort was provided by the Ethics Committee of Fuwai Hospital (2016‐847), and the guidelines of the Declaration of Helsinki were followed. Formal informed consent was acquired from every individual (version number AS2016‐1.1). Ethical approval for the UK Biobank research design was given by the North West Multicenter Research Ethics Committee (REC reference: 11/NW/0382), with explicit informed consent gathered from every volunteer.

## Conflicts of Interest

All authors declare no conflicts of interest.

## Supporting information




**Supporting File 1**: mco270786‐sup‐0001‐SuppMat.docx

## Data Availability

Datasets can be provided following a justified request to the primary investigators.
