# Supplementary material for: The Association Between Fibrosis‐4 Index and All‐Cause and Cardiac Mortality in Patients With Coronary Heart Disease Combined With Diabetes or Prediabetes: Findings From Two Large‐Scale Prospective Cohort Studies
Source: MedComm (2020). 2026 Jun 4;7(6):e70786. doi: 10.1002/mco2.70786 (PMC13238662; doi:10.1002/mco2.70786)
Supplement: Supplementary file 1 — Supporting File 1: mco270786‐sup‐0001‐SuppMat.docx [file MCO2-7-e70786-s001.docx]

**The association between fibrosis-4 index and all-cause and cardiac mortality in patients with coronary heart disease combined with diabetes or prediabetes: findings from two large-scale prospective cohort studies**

**Running title: FIB-4 and outcome in CHD with DM or preDM**

Chenxi Song^1,4#^, Zhihao Zheng^1,4#^, Xiaohui Bian^1,4^, Zheng Qiao^1,4^, Jiaxi Cheng^1,4^, Wanqing Sun^1,4^, Chunyue Wang^1,4^, Bowen Li^1,4^, Pengyu Liu^5^, Yuqin He^6^, Rui Fu^3,4*^ and Kefei Dou^1,2,4*^

^1^Department of Cardiology, Fuwai Hospital, Chinese Academy of Medical Sciences and Peking Union Medical College, Beijing, China.

^2^Cardiometabolic Medicine Center, Fuwai Hospital, Chinese Academy of Medical Sciences and Peking Union Medical College, Beijing, China.

^3^Department of Emergency, Fuwai Hospital, Chinese Academy of Medical Sciences and Peking Union Medical College, Beijing, China.

^4^State Key Laboratory of Cardiovascular Disease, National Center for Cardiovascular Diseases, Beijing, China.

^5^Emergency Department, Inner Mongolia Hospital of Traditional Chinese Medicine,

Hohhot, China

^6^Geriatric Department, Lanzhou First People's Hospital, Lanzhou, China

Correspondence to:

Kefei Dou, Cardiometabolic Medicine Center, Department of Cardiology, Fuwai Hospital, National Center for Cardiovascular Diseases, Chinese Academy of Medical Sciences and Peking Union Medical College, State Key Laboratory of Cardiovascular Disease, Beijing, China. A 167, Beilishi Road, Xicheng District, Beijing 100037, China. E-mail: drdoukefei@126.com and

Rui Fu, Department of Emergency, Fuwai Hospital, Chinese Academy of Medical Sciences and Peking Union Medical College, State Key Laboratory of Cardiovascular Disease, Beijing, China. A 167, Beilishi Road, Xicheng District, Beijing 100037, China. E-mail: fwfurui@163.com

Chenxi Song and Zhihao Zheng contributed equally to this work as first authors.

Kefei Dou and Rui Fu contributed equally to this work as corresponding authors.

**Figure Legends:**

**Figure S1.** Distribution of the FIB-4 index in the Fuwai Cohort.

FIB-4 = fibrosis-4 index.

**Figure S2.** Distribution of the FIB-4 index in the UK Biobank Cohort.

FIB-4 = fibrosis-4 index.

**Figure S3. Study flow chart in the UK Biobank Cohort.** A total of 8870 patients diagnosed with coronary heart disease combined with diabetes or prediabetes were screened. After the exclusion of 1699 patients with missing data on platelet count, ALT or AST, 723 patients with chronic liver disease or alcohol-related disease, and 770 patients with cancer, a total of 5678 patients were included for final analysis.

ALT = Alanine aminotransferase, AST= Aspartate aminotransferase, FIB-4=Fibrosis-4 index.

**Figure S4.** The graphical display of examining the proportionality assumption of each covariate used in the multivariate Cox regression model in the Fuwai Cohort.

**Figure S5.** The graphical display of examining the proportionality assumption of each covariate used in the multivariate Cox regression model in the UK Biobank Cohort.

**Table Legends:**

**Table S1.** Baseline characteristics according to baseline FIB-4 category in the UK biobank cohort.

**Table S2.** FIB-4 and mortality risk in the Fuwai Cohort after multivariable confounding adjustment.

**Table S3.** The association between FIB-4 with all-cause and cardiac mortality in the UK Biobank Cohort.

**Table S4.** FIB-4 and mortality risk in the UKB Cohort after multivariable confounding adjustment.

**Table S5.** Sensitivity analysis of the association between FIB-4 with mortality risk in the Fuwai Cohort.

**Table S6.** Sensitivity analysis of the association between FIB-4 with mortality risk in the UK Biobank Cohort.

**Table S7.** Sensitivity analysis using the WHO-criteria definition of prediabetes in the Fuwai Cohort.

**Table S8.** Sensitivity analysis using the WHO-criteria definition of prediabetes in the UK Biobank Cohort.

**Table S9.** The association between FIB-4 with mortality risk after excluding patients who died in the first 6 months in the Fuwai Cohort.

**Table S10.** The association between FIB-4 with mortality risk after excluding patients who died in the first 6 months in the UK Biobank Cohort.

**Table S11.** Subgroup analysis of the association between FIB-4 with cardiac mortality risk in the Fuwai Cohort.

**Table S12.** Subgroup analysis of the association between FIB-4 with cardiac mortality risk in the UK Biobank Cohort.

**Table S13.** Data identifier of key variables.

**Table S14.** The missing proportion of covariates in the Fuwai and UK Biobank Cohort.


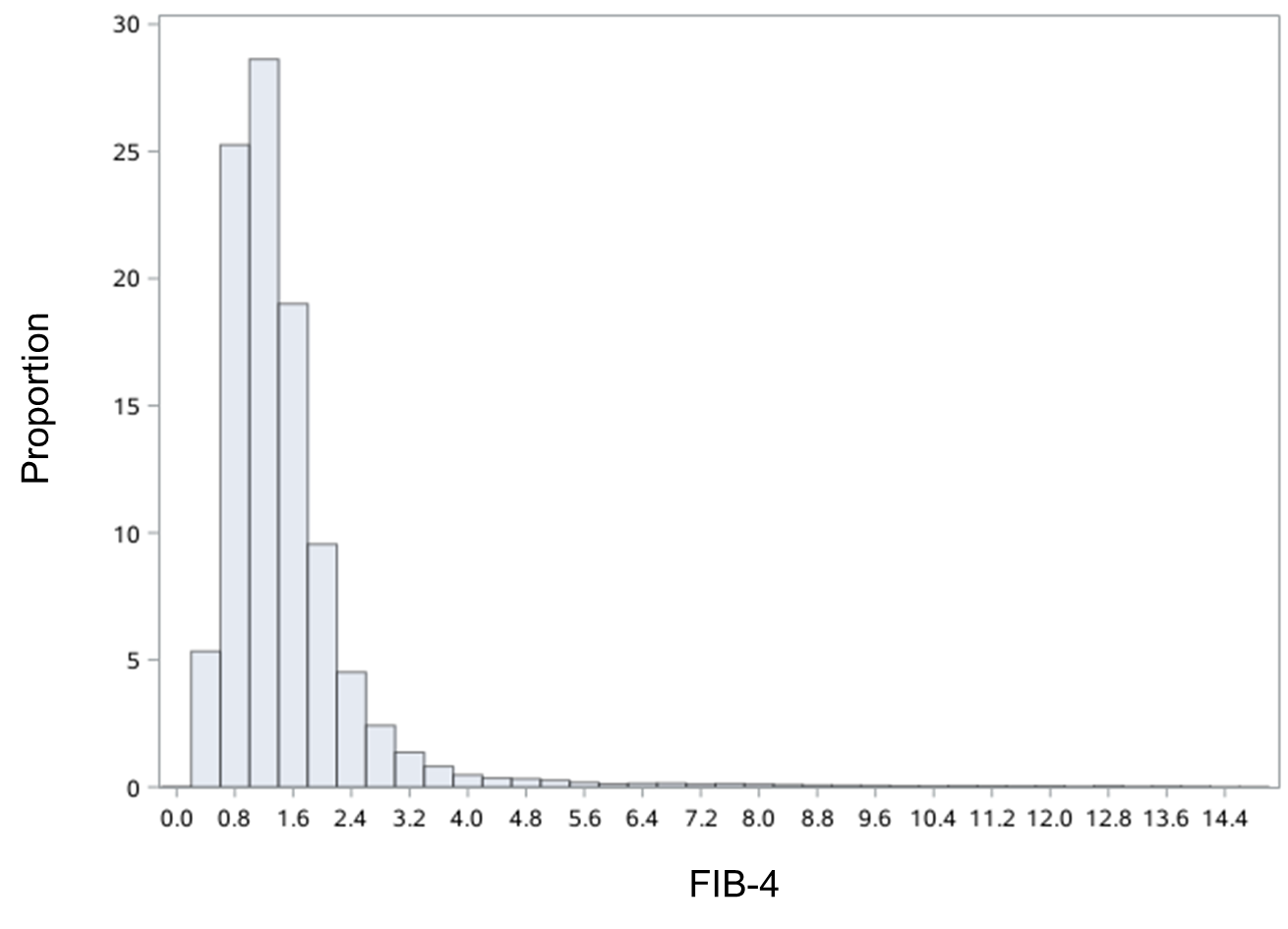


**Figure S1.** Distribution of the FIB-4 index in the Fuwai Cohort.

FIB-4 = fibrosis-4 index.


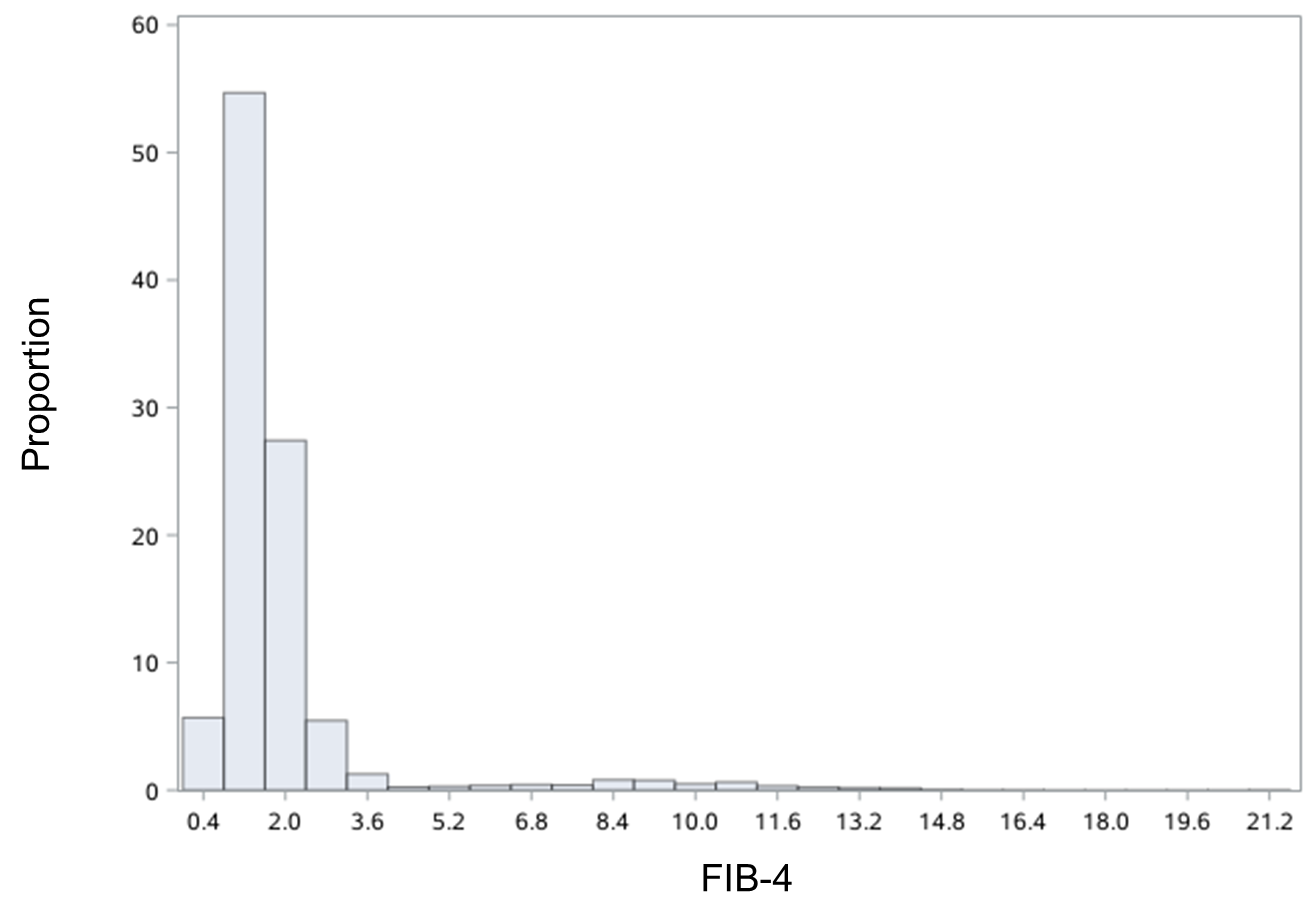


**Figure S2.** Distribution of the FIB-4 index in the UK Biobank Cohort.

FIB-4 = fibrosis-4 index.


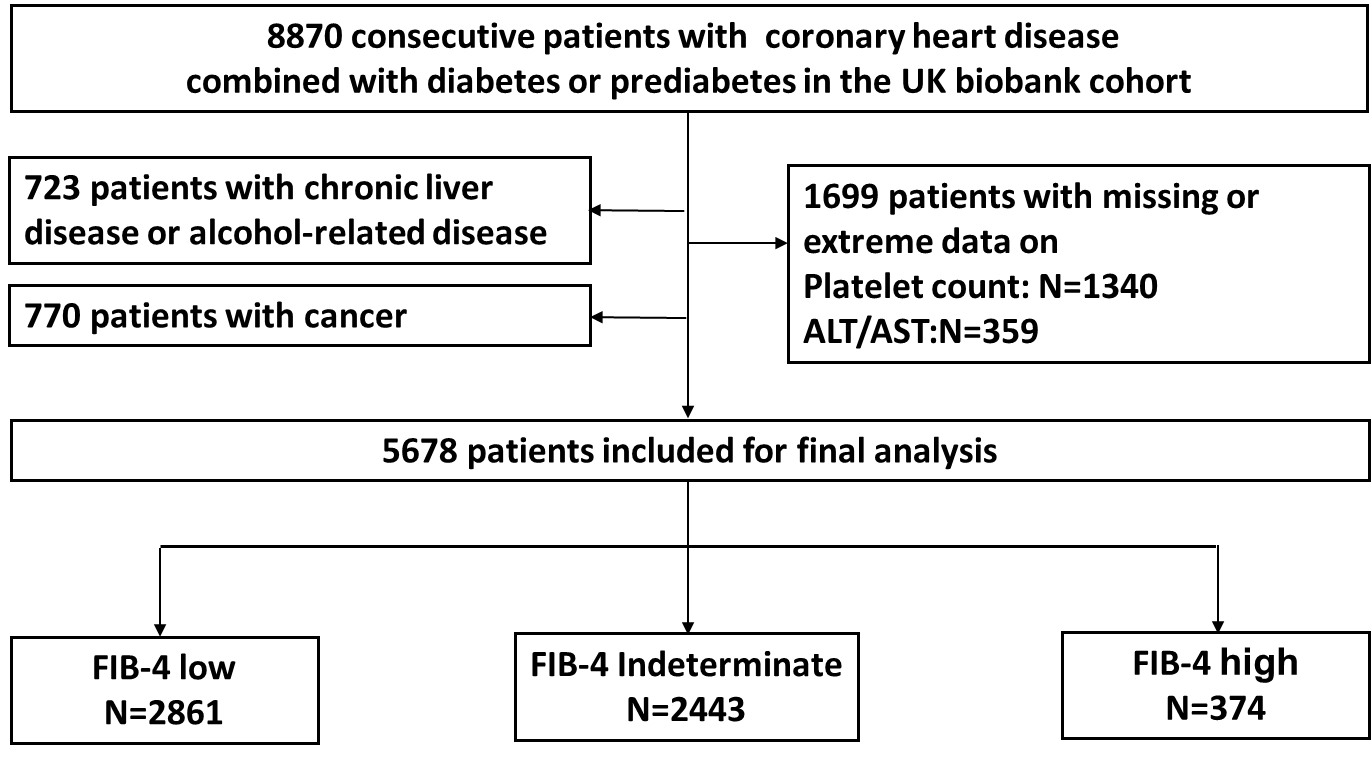


**Figure S3. Study flow chart in the UK Biobank Cohort.** A total of 8870 patients diagnosed with coronary heart disease combined with diabetes or prediabetes were screened. After the exclusion of 1699 patients with missing data on platelet count, ALT or AST, 723 patients with chronic liver disease or alcohol-related disease, and 770 patients with cancer, a total of 5678 patients were included for final analysis.

ALT = Alanine aminotransferase, AST= Aspartate aminotransferase, FIB-4=Fibrosis-4 index.


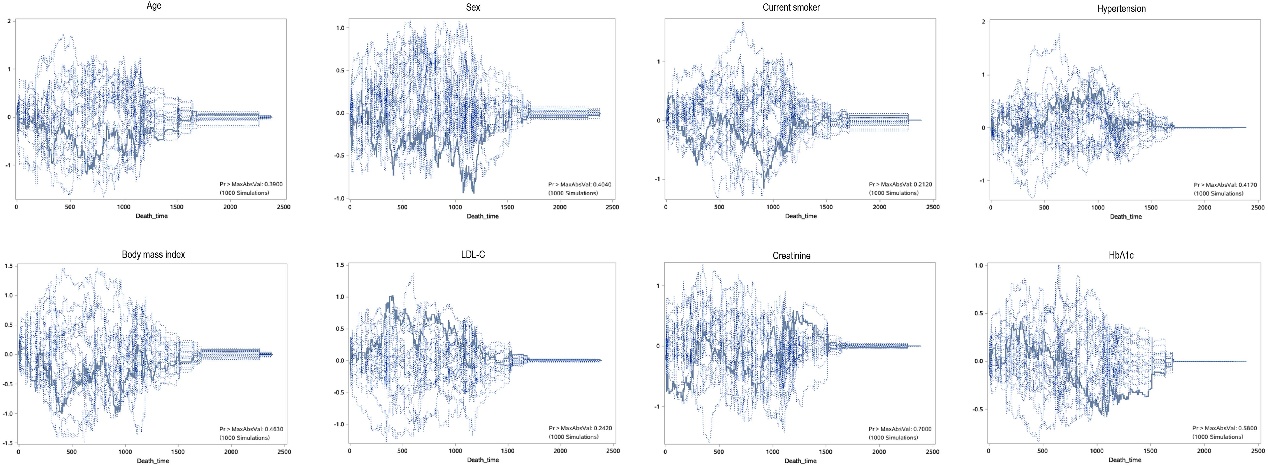


**Figure S4.** The graphical display of examining the proportionality assumption of each covariate used in the multivariate Cox regression model in the Fuwai Cohort.


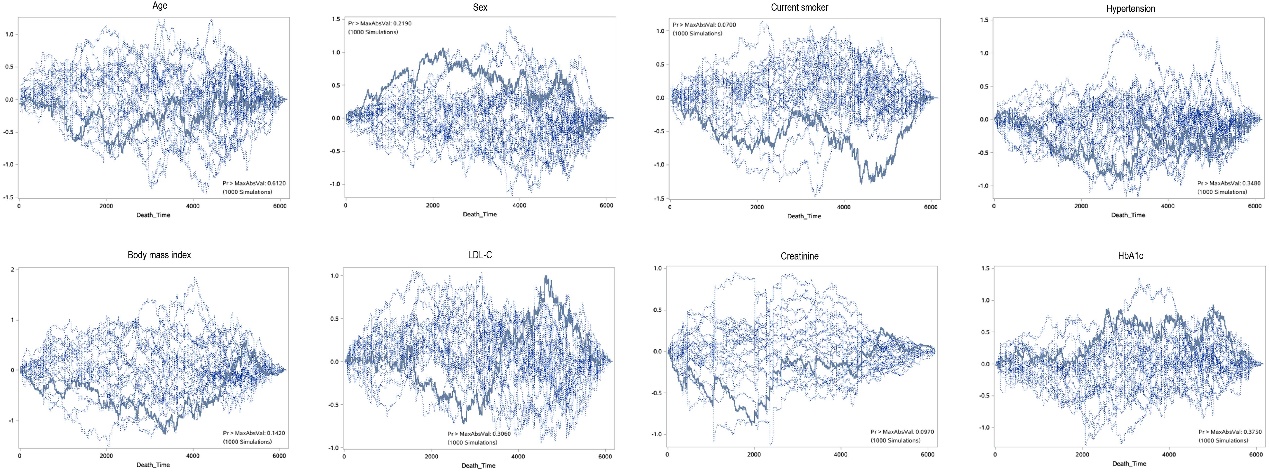


**Figure S5.** The graphical display of examining the proportionality assumption of each covariate used in the multivariate Cox regression model in the UK Biobank Cohort.

**Table S1. Baseline characteristics according to baseline FIB-4 category in the UK biobank cohort**

| **Characteristics** | **FIB-4 low**  **N=2861** | **FIB-4** **indeterminate**  **N=2443** | **FIB-4 high**  **N=374** | **P value** |
| --- | --- | --- | --- | --- |
| Age, years | 60.59±6.22 | 63.94±4.49 | 62.41±6.07 | <0.0001 |
| Male, n (%) | 1867/2861 (65.26) | 1847/2443 (75.60) | 241/374 (64.44) | <0.0001 |
| BMI, kg/m^2^ | 31.43±5.67 | 30.22±5.19 | 30.74±5.83 | <0.0001 |
| Current smokers, n (%) | 383/2857 (13.41) | 217/2442 (8.89) | 42/374 (11.23) | <0.0001 |
| Hypertension, n (%) | 1845/2861 (64.49) | 1576/2443 (64.51) | 248/374 (66.31) | 0.7781 |
| SBP, mmHg | 138 (124, 151) | 140 (126, 154) | 139 (127, 153) | 0.0612 |
| DBP, mmHg | 78 (70, 85) | 77 (69, 84) | 78 (70, 86) | 0.0472 |
| Prior MI, n (%) | 1218/2861 (42.57) | 1111/2443 (45.48) | 174/374 (46.52) | 0.0646 |
| Prior Stroke, n(%) | 198/2861 (6.92) | 181/2443 (7.41) | 28/374 (7.49) | 0.7660 |
| Biochemical parameters |  |  |  |  |
| ALT(U/L) | 24.60 (18.76, 32.20) | 23.75 (17.75, 32.58) | 23.48 (18.27, 32.93) | 0.1507 |
| AST(U/L) | 23.90 (20.30, 28.30) | 28.30 (24.00, 34.80) | 26.70 (22.50, 34.20) | <0.0001 |
| PLT(10^9^/L) | 265.10 (237.0, 298.4) | 198.0 (175.80, 235.30) | 36.0 (33.0, 49.90) | <0.0001 |
| TC, mmol/L | 4.52±1.07 | 4.37±1.01 | 4.53±1.12 | <0.0001 |
| HDL-C, mmol/L | 1.23±0.36 | 1.27±0.38 | 1.28±0.37 | 0.0002 |
| LDL-C, mmol/L | 2.74±0.77 | 2.62 ± 0.71 | 2.73±0.79 | <0.0001 |
| TG, mmol/L | 1.95 (1.45, 2.79) | 1.79 (1.31, 2.60) | 1.84 (1.39, 2.57) | <0.0001 |
| Lp(a), mg/L | 17.61 (7.50, 57.41) | 17.10 (7.50, 59.99) | 18.79 (8.90, 63.23) | 0.5415 |
| ApoA, g/L | 1.41±0.26 | 1.43±0.27 | 1.46±0.28 | 0.0004 |
| ApoB, g/L | 0.92±0.30 | 0.87±0.28 | 0.9±0.29 | <0.0001 |
| FPG, mmol/L | 6.84±2.78 | 6.66±2.45 | 6.78±2.56 | 0.0654 |
| HbA1c, % | 4.82±1.37 | 4.58±1.22 | 4.75±1.31 | <0.0001 |
| CRP, mg/L | 2.04 (0.96, 4.28) | 1.60 (0.80, 3.17) | 2.20 (1.05, 4.75) | <0.0001 |
| Creatinine, μmol/L | 75.30 (65.10, 87.55) | 79.40 (69.00, 92.25) | 78.00 (67.90, 90.40) | <0.0001 |
| Medication at discharge | |  |  |  |
| Antidiabetic drugs, n (%) | 1201/2861(41.98) | 816/2443 (33.40) | 131/374 (35.03) | <0.0001 |
| Lipid-lowering drugs, n(%) | 1634/2861 (57.11) | 1649/2443 (67.50) | 219/374 (58.56) | <0.0001 |

FIB-4 index was categorized as low (<1.45), indeterminate (1.45–3.25), and high (>3.25).

FIB-4 = fibrosis-4 index; BMI = body mass index; SBP = systolic blood pressure; DBP = diastolic blood pressure; MI = myocardial infarction; ALT=Alanine Aminotransferase; AST= Aspartate Aminotransferase; PLT = platelet; TC = total cholesterol; HDL-C = high-density lipoprotein cholesterol; LDL-C = low-density lipoprotein cholesterol; TG = triglycerides; Lp(a) = Lipoprotein (a); ApoA= Apolipoprotein A; ApoB = Apolipoprotein B; FPG = fasting plasma glucose; HbA1c = glycated haemoglobin; CRP = C-reactive protein;

**Table S2.** **FIB-4 and mortality risk in the Fuwai Cohort after multivariable confounding adjustment**

|  | **Event/Total (%)** | **Model 1** | **P value** | **Model 2** | **P value** |
| --- | --- | --- | --- | --- | --- |
| **All-cause mortality** |  |  |  |  |  |
| **FIB-4 low** | 208/12479 (1.67) | 1 (ref) | 1 (ref) | 1 (ref) | 1 (ref) |
| **FIB-4 indeterminate** | 245/6731 (3.64) | 1.145 (0.920, 1.425) | 0.2241 | 1.139 (0.915, 1.417) | 0.2443 |
| **FIB-4 high** | 89/923 (9.64) | 2.413 (1.801, 3.232) | <.0001 | 1.898 (1.416, 2.545) | <.0001 |
| **FIB-4 per one unit increase** | 542/20133 (2.69) | 1.031 (1.017, 1.045) | <.0001 | 1.022 (1.006, 1.038) | 0.0056 |
| **Cardiac mortality** |  |  |  |  |  |
| **FIB-4 low** | 79/12479(0.63) | 1 (ref) | 1 (ref) | 1 (ref) | 1 (ref) |
| **FIB-4 indeterminate** | 95/6731(1.41) | 1.243 (0.897, 1.721) | 0.1908 | 1.107 (0.759, 1.613) | 0.5977 |
| **FIB-4 high** | 36/923(3.90) | 2.614 (1.677, 4.072) | <.0001 | 2.001 (1.241, 3.226) | 0.0044 |
| **FIB-4 per one unit increase** | 210/20133(1.04) | 1.037 (1.017, 1.056) | 0.0001 | 1.029 (1.007, 1.052) | 0.0110 |

Model1 adjusted for age, sex, current smoking status, prior hypertension, body mass index, low‐density lipoprotein

cholesterol, eGFR, HbA1c, statins and prior MI. Model 2 adjusted for the confounders in model 1 plus LVEF

and triple vessel disease

FIB-4 index was categorized as low (<1.45), indeterminate (1.45–3.25), and high (>3.25).

**Table S3.** **The association between FIB-4 with all-cause and cardiac mortality in the UK Biobank Cohort**

|  | **Event/Total (%)** | **Crude HR** | **P value** | **Adjusted HR** | **P value** |
| --- | --- | --- | --- | --- | --- |
| **All-cause mortality** |  |  |  |  |  |
| **FIB-4 low** | 917/2861 (32.05) | 1 (ref) | 1 (ref) | 1 (ref) | 1 (ref) |
| **FIB-4 indeterminate** | 864/2443 (35.57) | 1.136 (1.035, 1.247) | 0.0070 | 0.978 (0.885, 1.082) | 0.6663 |
| **FIB-4 high** | 152/374 (40.64) | 1.368 (1.152, 1.624) | 0.0004 | 1.261 (1.058, 1.504) | 0.0098 |
| **FIB-4 per one unit increase** | 1933/5678 (34.04) | 1.033 (1.011, 1.055) | 0.0025 | 1.024 (1.001, 1.047) | 0.0410 |
| **Cardiac mortality** |  |  |  |  |  |
| **FIB-4 low** | 357/2861 (12.48) | 1 (ref) | 1 (ref) | 1 (ref) | 1 (ref) |
| **FIB-4 indeterminate** | 356/2443 (14.57) | 1.203 (1.039 1.393) | 0.0137 | 1.007 (0.860, 1.179) | 0.9321 |
| **FIB-4 high** | 69/374 (18.45) | 1.589 (1.228, 2.056) | 0.0004 | 1.407 (1.079, 1.836) | 0.0118 |
| **FIB-4 per one unit increase** | 782/5678 (13.77) | 1.048 (1.016, 1.081) | 0.0033 | 1.035 (1.001, 1.071) | 0.0421 |

Adjusted for age, sex, current smoking status, prior hypertension, body mass index, low‐density lipoprotein

cholesterol, eGFR and HbA1c.

FIB-4 index was categorized as low (<1.45), indeterminate (1.45–3.25), and high (>3.25).

**Table S4. FIB-4 and mortality risk in the UKB Cohort after multivariable confounding adjustment**

|  | **Event/Total (%)** | **Model 1** | **P value** | **Model 2** | **P value** |
| --- | --- | --- | --- | --- | --- |
| **All-cause mortality** |  |  |  |  |  |
| **FIB-4 low** | 917/2861 (32.05) | 1 (ref) | 1 (ref) | 1 (ref) | 1 (ref) |
| **FIB-4 indeterminate** | 864/2443 (35.57) | 0.973 (0.880, 1.076) | 0.5969 | 0.980 (0.886, 1.083) | 0.6890 |
| **FIB-4 high** | 152/374 (40.64) | 1.249 (1.048, 1.490) | 0.0132 | 1.229 (1.031, 1.467) | 0.0218 |
| **FIB-4 per one unit increase** | 1933/5678 (34.04) | 1.023 (1.000, 1.046) | 0.0509 | 1.021 (0.998, 1.044) | 0.0735 |
| **Cardiac mortality** |  |  |  |  |  |
| **FIB-4 low** | 357/2861 (12.48) | 1 (ref) | 1 (ref) | 1 (ref) | 1 (ref) |
| **FIB-4 indeterminate** | 356/2443 (14.57) | 0.998 (0.853, 1.168) | 0.9801 | 1.004 (0.858, 1.175) | 0.9622 |
| **FIB-4 high** | 69/374 (18.45) | 1.379 (1.057, 1.799) | 0.0178 | 1.350 (1.034, 1.762) | 0.0274 |
| **FIB-4 per one unit increase** | 782/5678 (13.77) | 1.033 (0.999, 1.068) | 0.0579 | 1.031 (0.996, 1.066) | 0.0793 |

Model1 adjusted for age, sex, current smoking status, prior hypertension, body mass index, low‐density lipoprotein

cholesterol, eGFR, HbA1c, statins, prior MI. Model 2 adjusted for the confounders in model 1 plus alcohol

intake, hepatotoxic medications

FIB-4 index was categorized as low (<1.45), indeterminate (1.45–3.25), and high (>3.25).

**Table S5. Sensitivity analysis of the association between FIB-4 with mortality risk in the Fuwai Cohort^a^**

|  | **Event/Total (%)** | **Crude HR** | **P value** | **Adjusted HR** | **P value** |
| --- | --- | --- | --- | --- | --- |
| **All-cause mortality** |  |  |  |  |  |
| **FIB-4 low** | 175/10575(1.65) | 1 (ref) | 1 (ref) | 1 (ref) | 1 (ref) |
| **FIB-4 indeterminate** | 253/8089(3.13) | 1.902 (1.568, 2.307) | <0.0001 | 1.016 (0.823, 1.253) | 0.8851 |
| **FIB-4 high** | 114/1469(7.76) | 5.003 (3.950, 6.337) | <0.0001 | 1.854 (1.414, 2.430) | <0.0001 |
| **FIB-4 per one unit increase** | 542/20133(2.69) | 1.082 (1.072, 1.093) | <0.0001 | 1.033 (1.020, 1.047) | <0.0001 |
| **Cardiac mortality** |  |  |  |  |  |
| **FIB-4 low** | 68/10575(0.64) | 1 (ref) | 1 (ref) | 1 (ref) | 1 (ref) |
| **FIB-4 indeterminate** | 97/8089(1.20) | 1.854 (1.360, 2.528) | <0.0001 | 1.006 (0.719, 1.408) | 0.9708 |
| **FIB-4 high** | 45/1469(3.06) | 5.068 (3.477, 7.388) | <0.0001 | 1.988 (1.292, 3.060) | 0.0018 |
| **FIB-4 per one unit increase** | 210/20133(1.04) | 1.086 (1.071, 1.101) | <0.0001 | 1.041 (1.022, 1.061) | <0.0001 |

Adjusted for age, sex, current smoking status, prior hypertension, body mass index, low‐density lipoprotein

cholesterol, eGFR and HbA1c.

^a^FIB-4 index was categorized as low (<1.30), indeterminate (1.30–2.67), and high (>2.67).

**Table S6. Sensitivity analysis of the association between FIB-4 with mortality risk in the UK Biobank Cohort^a^**

|  | **Event/Total (%)** | **Crude HR** | **P value** | **Adjusted HR** | **P value** |
| --- | --- | --- | --- | --- | --- |
| **All-cause mortality** |  |  |  |  |  |
| **FIB-4 low** | 712/2218 (32.10) | 1 (ref) | 1 (ref) | 1 (ref) | 1 (ref) |
| **FIB-4 indeterminate** | 1008/2941 (34.27) | 1.092 (0.992, 1.202) | 0.0739 | 0.914 (0.824, 1.014) | 0.0900 |
| **FIB-4 high** | 213/519 (41.04) | 1.356 (1.163, 1.580) | <0.0001 | 1.231 (1.050, 1.443) | 0.0103 |
| **FIB-4 per one unit increase** | 1933/5678 (34.04) | 1.033 (1.011, 1.055) | 0.0025 | 1.024 (1.001, 1.047) | 0.0410 |
| **Cardiac mortality** |  |  |  |  |  |
| **FIB-4 low** | 269/2218 (12.13) | 1 (ref) | 1 (ref) | 1 (ref) | 1 (ref) |
| **FIB-4 indeterminate** | 418/2941 (14.21) | 1.197 (1.027, 1.396) | 0.0215 | 0.975 (0.827, 1.150) | 0.7648 |
| **FIB-4 high** | 95/519 (18.30) | 1.600 (1.266, 2.023) | <0.0001 | 1.392 (1.092, 1.776) | 0.0077 |
| **FIB-4 per one unit increase** | 782/5678 (13.77) | 1.048 (1.016, 1.081) | 0.0033 | 1.035 (1.001, 1.071) | 0.0421 |

Adjusted for age, sex, current smoking status, prior hypertension, body mass index, low‐density lipoprotein

cholesterol, eGFR and HbA1c.

^a^FIB-4 index was categorized as low (<1.30), indeterminate (1.30–2.67), and high (>2.67).

**Table S7. Sensitivity analysis using the WHO-criteria definition of prediabetes** **in the Fuwai Cohort**

|  | **Event/Total (%)** | **Crude HR** | **P value** | **Adjusted HR** | **P value** |
| --- | --- | --- | --- | --- | --- |
| **All-cause mortality** |  |  |  |  |  |
| **FIB-4 low** | 162/8694(1.86) | 1 (ref) | 1 (ref) | 1 (ref) | 1 (ref) |
| **FIB-4** **indeterminate** | 186/4702(3.96) | 2.154 (1.744, 2.660) | <0.0001 | 1.217 (0.968, 1.529) | 0.0923 |
| **FIB-4 high** | 85/783(10.86) | 6.575 (5.054, 8.556) | <0.0001 | 2.859 (2.127, 3.844) | <0.0001 |
| **FIB-4 per one unit increase** | 433/14179(3.05) | 1.079 (1.069, 1.090) | <0.0001 | 1.034 (1.020, 1.047) | <0.0001 |
| **Cardiac mortality** |  |  |  |  |  |
| **FIB-4 low** | 63/8694(0.72) | 1 (ref) | 1 (ref) | 1 (ref) | 1 (ref) |
| **FIB-4 indeterminate** | 69/4702(1.47) | 2.037 (1.447, 2.866) | <0.0001 | 1.179 (0.816, 1.704) | 0.3798 |
| **FIB-4 high** | 35/783(4.47) | 7.054 (4.662, 10.67) | <0.0001 | 3.254 (2.040, 5.189) | <0.0001 |
| **FIB-4 per one unit increase** | 167/14179(1.18) | 1.083 (1.068, 1.098) | <0.0001 | 1.042 (1.022, 1.062) | <0.0001 |

Adjusted for age, sex, current smoking status, prior hypertension, body mass index, low‐density lipoprotein cholesterol, eGFR and HbA1c.

FIB-4 index was categorized as low (<1.45), indeterminate (1.45–3.25), and high (>3.25).

**Table S8. Sensitivity analysis using the WHO-criteria definition of prediabetes in the UK Biobank Cohort**

|  | **Event/Total (%)** | **Crude HR** | **P value** | **Adjusted HR** | **P value** |
| --- | --- | --- | --- | --- | --- |
| **All-cause mortality** |  |  |  |  |  |
| **FIB-4 low** | 756/2144 (35.26) | 1 (ref) | 1 (ref) | 1 (ref) | 1 (ref) |
| **FIB-4 indeterminate** | 684/1741 (39.29) | 1.147 (1.034, 1.272) | 0.0095 | 0.988 (0.884, 1.105) | 0.8341 |
| **FIB-4 high** | 121/271 (44.65) | 1.362 (1.124, 1.650) | 0.0016 | 1.241 (1.018, 1.512) | 0.0330 |
| **FIB-4 per one unit increase** | 1561/4156 (37.56) | 1.034 (1.010, 1.058) | 0.0052 | 1.022 (0.997, 1.049) | 0.0833 |
| **Cardiac mortality** |  |  |  |  |  |
| **FIB-4 low** | 289/2144 (13.48) | 1 (ref) | 1 (ref) | 1 (ref) | 1 (ref) |
| **FIB-4 indeterminate** | 280/1741 (16.08) | 1.230 (1.044, 1.450) | 0.0136 | 1.031 (0.865, 1.230) | 0.7307 |
| **FIB-4 high** | 54/271 (19.93) | 1.586 (1.186, 2.121) | 0.0019 | 1.383 (1.022, 1.871) | 0.0355 |
| **FIB-4 per one unit increase** | 623/4156 (14.99) | 1.043 (1.007, 1.081) | 0.0204 | 1.028 (0.988, 1.068) | 0.1714 |

Adjusted for age, sex, current smoking status, prior hypertension, body mass index, low‐density lipoprotein

cholesterol, eGFR and HbA1c.

FIB-4 index was categorized as low (<1.45), indeterminate (1.45–3.25), and high (>3.25).

**Table S9. The association between FIB-4 with mortality risk after excluding patients who died in the**

**first 6 months in the Fuwai Cohort**

|  | **Event/Total (%)** | **Crude HR** | **P value** | **Adjusted HR** | **P value** |
| --- | --- | --- | --- | --- | --- |
| **All-cause mortality** |  |  |  |  |  |
| **FIB-4 low** | 185/12362 (1.50) | 1 (ref) | 1 (ref) | 1 (ref) | 1 (ref) |
| **FIB-4 indeterminate** | 214/6645 (3.22) | 2.175 (1.786, 2.649) | <0.0001 | 1.156 (0.931, 1.435) | 0.1887 |
| **FIB-4 high** | 74/896 (8.26) | 6.073 (4.636, 7.957) | <0.0001 | 2.416 (1.786, 3.268) | <0.0001 |
| **FIB-4 per one unit increase** | 473/19903 (2.38) | 1.079 (1.067, 1.092) | <0.0001 | 1.029 (1.014, 1.045) | 0.0002 |
| **Cardiac mortality** |  |  |  |  |  |
| **FIB-4 low** | 69/12362 (0.56) | 1 (ref) | 1 (ref) | 1 (ref) | 1 (ref) |
| **FIB-4 indeterminate** | 81/6645 (1.22) | 2.181 (1.582, 3.007) | <0.0001 | 1.186 (0.834, 1.685) | 0.3420 |
| **FIB-4 high** | 30/896 (3.35) | 6.688 (4.354, 10.27) | <0.0001 | 2.876 (1.778, 4.652) | <0.0001 |
| **FIB-4 per one unit increase** | 180/19903 (0.90) | 1.083 (1.066, 1.100) | <0.0001 | 1.039 (1.018, 1.062) | 0.0003 |

Adjusted for age, sex, current smoking status, prior hypertension, body mass index, low‐density lipoprotein

cholesterol, eGFR and HbA1c.

FIB-4 index was categorized as low (<1.45), indeterminate (1.45–3.25), and high (>3.25).

**Table S10. The association between FIB-4 with mortality risk after excluding patients who died in the**

**first 6 months in the UK Biobank Cohort**

|  | **Event/Total (%)** | **Crude HR** | **P value** | **Adjusted HR** | **P value** |
| --- | --- | --- | --- | --- | --- |
| **All-cause mortality** |  |  |  |  |  |
| **FIB-4 low** | 910/2853 (31.90) | 1 (ref) | 1 (ref) | 1 (ref) | 1 (ref) |
| **FIB-4 indeterminate** | 849/2427 (34.98) | 1.126 (1.025, 1.236) | 0.0132 | 0.969 (0.876, 1.072) | 0.5418 |
| **FIB-4 high** | 151/372 (40.59) | 1.370 (1.153, 1.628) | 0.0003 | 1.264 (1.059, 1.508) | 0.0094 |
| **FIB-4 per one unit increase** | 1910/5652 (33.79) | 1.033 (1.011, 1.055) | 0.0029 | 1.023 (1.001, 1.047) | 0.0442 |
| **Cardiac mortality** |  |  |  |  |  |
| **FIB-4 low** | 351/2853 (12.30) | 1 (ref) | 1 (ref) | 1 (ref) | 1 (ref) |
| **FIB-4 indeterminate** | 348/2427 (14.34) | 1.197 (1.032, 1.388) | 0.0178 | 0.997 (0.851, 1.170) | 0.9746 |
| **FIB-4 high** | 68/372 (18.28) | 1.595 (1.230, 2.068) | 0.0004 | 1.410 (1.079, 1.844) | 0.0119 |
| **FIB-4 per one unit increase** | 767/5652 (13.57) | 1.048 (1.015, 1.081) | 0.0039 | 1.035 (1.000, 1.071) | 0.0481 |

Adjusted for age, sex, current smoking status, prior hypertension, body mass index, low‐density lipoprotein

cholesterol, eGFR and HbA1c.

FIB-4 index was categorized as low (<1.45), indeterminate (1.45–3.25), and high (>3.25).

**Table S11. Subgroup analysis of the association between FIB-4 with cardiac mortality risk in the Fuwai Cohort**

|  | **Event/Total (%)** | **Crude HR** | **P value** | **Adjusted HR** | **P value** | **P _interaction_** |
| --- | --- | --- | --- | --- | --- | --- |
| **Diabetes (N=10840)** |  |  |  |  |  | **0.3021** |
| FIB-4 low | 44/6668 (0.66) | 1 (ref) | 1 (ref) | 1 (ref) | 1 (ref) |  |
| FIB-4 indeterminate | 50/3490 (1.43) | 2.206 (1.471, 3.307) | 0.0001 | 1.291 (0.837, 1.992) | 0.2477 |  |
| FIB-4 high | 31/682 (4.55) | 7.993 (5.042, 12.67) | <0.0001 | 3.575 (2.123, 6.019) | <0.0001 |  |
| **Prediabetes (N=9293)** |  |  |  |  |  |  |
| FIB-4 low | 35/5811 (0.60) | 1 (ref) | 1 (ref) | 1 (ref) | 1 (ref) |  |
| FIB-4 indeterminate | 45/3241 (1.39) | 2.249 (1.443, 3.506) | 0.0003 | 1.184 (0.721, 1.947) | 0.5043 |  |
| FIB-4 high | 5/241 (2.07) | 3.616 (1.417, 9.231) | 0.0072 | 1.622 (0.602, 4.373) | 0.3390 |  |
| **Age ≥ 65 years (N=6704)** |  |  |  |  |  | 0.1386 |
| FIB-4 low | 31/6704 (1.25) | 1 (ref) | 1 (ref) | 1 (ref) | 1 (ref) |  |
| FIB-4 indeterminate | 74/3697 (2.00) | 1.588 (1.044, 2.416) | 0.0307 | 1.256 (0.815, 1.937) | 0.3017 |  |
| FIB-4 high | 26/524 (4.96) | 4.447 (2.638, 7.495) | <0.0001 | 2.356 (1.339, 4.145) | 0.0029 |  |
| **Age < 65 years (N=13429)** |  |  |  |  |  |  |
| FIB-4 low | 48/9996 (0.48) | 1 (ref) | 1 (ref) | 1 (ref) | 1 (ref) |  |
| FIB-4 indeterminate | 21/3034 (0.69) | 1.451 (0.869, 2.423) | 0.1549 | 1.118 (0.652, 1.916) | 0.6863 |  |
| FIB-4 high | 10/399 (2.51) | 5.700 (2.882, 11.27) | <0.0001 | 4.995 (2.460, 10.14) | <0.0001 |  |
| **Male (N=14836)** |  |  |  |  |  | 0.3835 |
| FIB-4 low | 58/9501 (0.61) | 1 (ref) | 1 (ref) | 1 (ref) | 1 (ref) |  |
| FIB-4 indeterminate | 72/4670 (1.54) | 2.530 (1.789, 3.580) | <0.0001 | 1.524 (1.041, 2.231) | 0.0304 |  |
| FIB-4 high | 23/665 (3.46) | 6.314 (3.894, 10.24) | <0.0001 | 3.097 (1.810, 5.301) | <0.0001 |  |
| **Female (N=5297)** |  |  |  |  |  |  |
| FIB-4 low | 21/2978 (0.71) | 1 (ref) | 1 (ref) | 1 (ref) | 1 (ref) |  |
| FIB-4 indeterminate | 23/2061 (1.12) | 1.585 (0.877, 2.863) | 0.1272 | 0.709 (0.377, 1.335) | 0.2874 |  |
| FIB-4 high | 13/258 (5.04) | 7.950 (3.977, 15.89) | <0.0001 | 2.488 (1.124, 5.508) | 0.0245 |  |
| **BMI≥25 kg/m^2^ (N=11870)** |  |  |  |  |  | 0.1284 |
| FIB-4 low | 46/7744 (0.59) | 1 (ref) | 1 (ref) | 1 (ref) | 1 (ref) |  |
| FIB-4 indeterminate | 35/3658 (0.96) | 1.609 (1.036, 2.497) | 0.0341 | 0.984 (0.606, 1.597) | 0.9484 |  |
| FIB-4 high | 13/468 (2.78) | 5.205 (2.810, 9.640) | <0.0001 | 3.097 (1.575, 6.090) | 0.0010 |  |
| **BMI<25 kg/m^2^ (N=8263)** |  |  |  |  |  |  |
| FIB-4 low | 33/4735 (0.7) | 1 (ref) | 1 (ref) | 1 (ref) | 1 (ref) |  |
| FIB-4 indeterminate | 60/3073 (1.95) | 2.804 (1.831, 4.294) | <0.0001 | 1.557 (0.984, 2.464) | 0.0586 |  |
| FIB-4 high | 23/455 (5.05) | 8.167 (4.793, 13.92) | <0.0001 | 2.863 (1.563, 5.245) | 0.0007 |  |
| **ACS (N=12528)** |  |  |  |  |  | 0.7946 |
| FIB-4 low | 51/7555 (0.68) | 1 (ref) | 1 (ref) | 1 (ref) | 1 (ref) |  |
| FIB-4 indeterminate | 58/4167 (1.39) | 2.065 (1.417, 3.009) | 0.0002 | 1.212 (0.807, 1.821) | 0.3549 |  |
| FIB-4 high | 31/806 (3.85) | 6.466 (4.134, 10.11) | <0.0001 | 3.195 (1.924, 5.307) | <0.0001 |  |
| **CCS (N=7605)** |  |  |  |  |  |  |
| FIB-4 low | 28/4924 (0.57) | 1 (ref) | 1 (ref) | 1 (ref) | 1 (ref) |  |
| FIB-4 indeterminate | 37/2564 (1.44) | 2.544 (1.557, 4.157) | 0.0002 | 1.287 (0.743, 2.229) | 0.3673 |  |
| FIB-4 high | 5/117 (4.27) | 7.695 (2.971, 19.93) | <0.0001 | 2.443 (0.865, 6.896) | 0.0917 |  |

Adjusted for age, sex, current smoking status, prior hypertension, body mass index, low‐density lipoprotein

cholesterol, eGFR and HbA1c.

FIB-4 index was categorized as low (<1.45), indeterminate (1.45–3.25), and high (>3.25).

**Table S12. Subgroup analysis of the association between FIB-4 with cardiac mortality risk in the UK Biobank Cohort**

|  | **Event/Total (%)** | **Crude HR** | **P value** | **Adjusted HR** | **P value** | **P _interaction_** |
| --- | --- | --- | --- | --- | --- | --- |
| **Diabetes (N=3382)** |  |  |  |  |  | **0.2587** |
| FIB-4 low | 265/1797 (14.75) | 1 (ref) | 1 (ref) | 1 (ref) | 1 (ref) |  |
| FIB-4 indeterminate | 253/1357 (18.64) | 1.317 (1.108, 1.564) | 0.0018 | 1.074 (0.892, 1.292) | 0.4518 |  |
| FIB-4 high | 47/228 (20.61) | 1.494 (1.095, 2.038) | 0.0112 | 1.281 (0.927, 1.771) | 0.1339 |  |
| **Prediabetes (N=2296)** |  |  |  |  |  |  |
| FIB-4 low | 92/1064 (8.65) | 1 (ref) | 1 (ref) | 1 (ref) | 1 (ref) |  |
| FIB-4 indeterminate | 103/1086 (9.84) | 1.127 (0.851, 1.493) | 0.4043 | 0.915 (0.677, 1.237) | 0.5634 |  |
| FIB-4 high | 22/146 (15.07) | 1.910 (1.199, 3.042) | 0.0065 | 1.808 (1.127, 2.901) | 0.0141 |  |
| **Age ≥ 65 years (N=2393)** |  |  |  |  |  | **0.0739** |
| FIB-4 low | 145/896 (16.18) | 1 (ref) | 1 (ref) | 1 (ref) | 1 (ref) |  |
| FIB-4 indeterminate | 218/1326 (16.44) | 1.025 (0.831, 1.266) | 0.8163 | 1.041 (0.835, 1.299) | 0.7208 |  |
| FIB-4 high | 47/171 (27.49) | 1.900 (1.367, 2.642) | 0.0001 | 1.834 (1.306, 2.574) | 0.0005 |  |
| **Age < 65 years (N=3285)** |  |  |  |  |  |  |
| FIB-4 low | 212/1965 (10.79) | 1 (ref) | 1 (ref) | 1 (ref) | 1 (ref) |  |
| FIB-4 indeterminate | 138/1117 (12.35) | 1.170 (0.944, 1.450) | 0.1512 | 1.010 (0.803, 1.271) | 0.9337 |  |
| FIB-4 high | 22/203 (10.84) | 1.028 (0.663, 1.595) | 0.9007 | 0.960 (0.605, 1.524) | 0.8638 |  |
| **Male (N=3955)** |  |  |  |  |  | 0.9600 |
| FIB-4 low | 283/1867 (15.16) | 1 (ref) | 1 (ref) | 1 (ref) | 1 (ref) |  |
| FIB-4 indeterminate | 307/1847 (16.62) | 1.146 (0.975, 1.348) | 0.0982 | 1.033 (0.869, 1.228) | 0.7156 |  |
| FIB-4 high | 53/241 (21.99) | 1.585 (1.182, 2.126) | 0.0021 | 1.406 (1.039, 1.902) | 0.0274 |  |
| **Female (N=1723)** |  |  |  |  |  |  |
| FIB-4 low | 74/994 (7.44) | 1 (ref) | 1 (ref) | 1 (ref) | 1 (ref) |  |
| FIB-4 indeterminate | 49/596 (8.22) | 1.059 (0.738, 1.520) | 0.7555 | 0.917 (0.624, 1.347) | 0.6580 |  |
| FIB-4 high | 16/133 (12.03) | 1.690 (0.984, 2.902) | 0.0570 | 1.612 (0.915, 2.842) | 0.0985 |  |
| **BMI≥25 kg/m^2^ (N=4982)** |  |  |  |  |  | 0.0993 |
| FIB-4 low | 331/2558 (12.94) | 1 (ref) | 1 (ref) | 1 (ref) | 1 (ref) |  |
| FIB-4 indeterminate | 309/2101 (14.71) | 1.179 (1.009, 1.377) | 0.0382 | 0.972 (0.824, 1.146) | 0.7327 |  |
| FIB-4 high | 59/323 (18.27) | 1.500 (1.137, 1.979) | 0.0041 | 1.316 (0.992, 1.745) | 0.0568 |  |
| **BMI<25 kg/m^2^ (N=696)** |  |  |  |  |  |  |
| FIB-4 low | 26/303 (8.58) | 1 (ref) | 1 (ref) | 1 (ref) | 1 (ref) |  |
| FIB-4 indeterminate | 47/342 (13.74) | 1.604 (0.994, 2.591) | 0.0531 | 1.372 (0.780, 2.413) | 0.2724 |  |
| FIB-4 high | 10/51 (19.61) | 2.605 (1.254, 5.412) | 0.0103 | 3.093 (1.323, 7.228) | 0.0091 |  |

Adjusted for age, sex, current smoking status, prior hypertension, body mass index, low‐density lipoprotein cholesterol, eGFR

and HbA1c.

FIB-4 index was categorized as low (<1.45), indeterminate (1.45–3.25), and high (>3.25).

**Table S13. Data identifier of key variables**

| **Variable** | **Identifier** |
| --- | --- |
| **Sociodemographic factor** |  |
| Age | Data-Field 21022 |
| Sex | Data-Field 31 |
| **Medical conditions** |  |
| Prior Angina | Data-Field 6150 |
| Prior Heart attack | Data-Field 6150 |
| Diabetes | Data-Field 2443 |
| Hypertension | Data-Field 6150 |
| **Medications** |  |
| Use of insulin | Data-Field 6177 |
| Other oral hypoglycemic medications^1^ | Data-Field 20003 |
| **Blood biochemistry** |  |
| Platelet count | Data-Field 30080 |
| Alanine aminotransferase | Data-Field 30620 |
| Aspartate aminotransferase | Data-Field 30650 |
| Total cholesterol | Data-Field 30690 |
| LDL-C | Data-Field 30780 |
| Triglycerides | Data-Field 30870 |
| Total bilirubin | Data-Field 30840 |
| Glucose | Data-Field 30740 |
| Glycated haemoglobin | Data-Field 30750 |
| Creatinine | Data-Field 30700 |
| C-reactive protein | Data-Field 30710 |
| **CHD definition and Death** |  |
| **ICD 10** | I20, I20.0, I20.1, I20.8, I20.9  I21, I21.0, I21.1, I21.2, I21.3, I21.4, I21.9,  I22, I22.0, I22.1, I22.8, I22.9, I23, I23.0, I23.1, I23.2, I23.3, I23.4, I23.5, I23.6, I23.8, I24, I24.0, I24.1, I24.8, I24.9, I25.1, I25.2, I25.5, I25.6, I25.8, I25.9 |
| **Cardiac Death** | **I00-I99** |

^1^Oral hypoglycemic medications include metformin, gliclazide, glipizide, rosiglitazone, pioglitazone and repaglinide.

**Table S14. The missing proportion of covariates in the Fuwai and UK Biobank Cohort**

|  | **Fuwai Cohort** | **UK Biobank** |
| --- | --- | --- |
| Age | 0/20133 (0%) | 0/5678 (0%) |
| Sex | 0/20133 (0%) | 0/5678 (0%) |
| Current_smoke | 11/20133 (0.1%) | 5/5678 (0.1%) |
| Hypertension | 5/20133 (0.02%) | 0/5678 (0%) |
| BMI | 444/20133 (2.2%) | 63/5678 (1.1%) |
| LDL_C | 1126/20133 (5.6%) | 10/5678 (0.2%) |
| Creatinine | 511/20133 (2.5%) | 4/5678 (0.1%) |
| HbA1c | 229/20133 (1.1%) | 293/5678 (5.2%) |
